# Incorporating time-resolved three-dimensional phase contrast (4D flow) MRI in clinical workflow: initial experiences at a large tertiary care medical center

**DOI:** 10.1186/1532-429X-15-S1-P32

**Published:** 2013-01-30

**Authors:** Bradley D Allen, Alex J Barker, Keyur Parekh, Lewis C Sommerville, Susanne Schnell, Kelly B Jarvis, Maria Carr, James Carr, Jeremy Collins, Michael Markl

**Affiliations:** 1 Radiology, Northwestern University Feinberg School of Medicine, Chicago, IL, USA

## Background

Time-resolved three-dimensional phase contrast (4D flow) MRI allows for visualization of three-dimensional cardiovascular anatomy and pulsatile flow with full volumetric coverage in a single, easy to prescribe 3D acquisition. The technique provides comprehensive flow visualization and permits retrospective flow quantification at any user-defined region of interest. [1] To our knowledge, no center has incorporated 4D flow MRI as a part of standard clinical cardiovascular MRI (CMR). The goals of this study include: 1) reporting on the incorporation of 4D flow MRI acquisition and processing as part of clinical CMR workflow and 2) better understanding the clinical impact of 3D flow visualization and retrospective flow quantification derived from 4D flow MRI in CMR.

## Methods

Patients referred to Northwestern Memorial Hospital for CMR with relevant clinical indications as judged by an attending radiologist were selected to have 4D flow MRI included in accordance with an IRB-approved protocol. Images were processed using in-house software for noise reduction, anti-aliasing, and eddy current correction. Flow visualization and quantification were performed using EnSight (CEI, Apex, NC). Processing time was recorded. Resulting 4D flow pathline cine images and flow quantification results were converted to DICOM format and transferred to the local PACS server to be reviewed as part of the patient's clinical images. Clinician-requested quantitative data was compared between 4D flow and two-dimensional phase contrast (2D PC) MRI techniques. Each case was retrospectively reviewed by an attending radiologist who assigned a qualitative measure of the 4D flow analysis' impact on the case 1) excluding 2D PC and 2) including 4D flow and 2D PC together (criteria listed in Table 1).

**Table 1:** Clinical cases with 4D flow imaging requests and results of analysis.

		Quantitative results					Subjective 4D Flow Impact Factor (scale: changed clinical impression = 4, quantitative information added = 3, relevant but did not impact case = 2, information not relevant to case = 1)	
		Clinical indication for 4D flow request	Quantitative comparison requested	4D results	2D PC results	Difference (%)	4D flow alone (excludes 2D PC data)	Case including both 2D PC and 4D flow

	1	Biscuspid aortic valve, flow pattern interest	Regurgitant fraction	16.57%	25%	34%	3	2
	2	Qp:Qs ratio in patient with VSD	Qsp:Qs ratio	1.22	Not measured	N/A	3	3
	3	Aortic stenosis, flow pattern interest	Aortic root peak velocity	3.52 m/s	3.8 m/s	7%	2	3
	4	Biscuspid valve, flow pattern interest	Aortic root peak velocity	2.35 m/s	Note measured	N/A	3	2
Valvular	5	Biscuspid valve, flow pattern interest, aortic regurgitation. Regurgitant jet is very eccentric, limiting 2D assessment	Regurgitant fraction	13.36%	57%	77%	2	3
	6	Biscuspid aortic valve, flow pattern interest, aortic regurgitation and stenosis assessment	Regurgitant fraction	8.75%	34%	74%	2	3
	7	Aortic root velocity, 2D PC data underestimated velocity	Aortic root peak velocity	4.08 m/s	3.5 m/s	17%	4	4

	8	Bicuspid aortic valve, flow pattern interest, aortic regurgitation and stenosis assessment	Regurgitant fraction	4.66%	15%	69%	3	2
Aneurysm	9	Flow pattern interest, aortic dilation	Ascending aorta peak velocity	1.44 m/s	1.3 m/s	11%	3	2
	10	Aortic stenosis and regurgitation assessment	Regurgitant fraction	38%	55%	31%	3	3
			Aortic root peak velocity	3.36 m/s	3.8 m/s	12%		
	11	Aortic stenosis and regurgitation assessment	Aortic root peak velocity	3.89 m/s	4.0 m/s	5%	4	4

	12	Flow pattern interest, split flow right-left PA	Peak velocity through pulmonary anastomis	2.32 m/s	1.4 m/s	66%	4	4
			Peak velocity through pulmonary post-stenosis	2.70 m/s	2.5 m/s	8%		
Post-surgical	13	Aortic valve assessment	Ascending aorta peak velocity	Could not measure velocity secondary to aliasing artifact	Not measured	N/A	Not available	Not available
	14	Aortic stenosis and regurgitation assessment	Regurgitant fraction	4.53%	Data limited by artifact	N/A	3	3
			Aortic root peak velocity	3.11 m/s	Data limited by artifact	N/A		

Congenital	15	Large pulmonary regurgitant fraction by 2D PC	Pulmonary valve regurgitant fraction	6.41%	21.5%	70%	1	2
	16	Flow pattern, Qp:Qs	Qp:Qs Ratio	1.08	1	8%	3	3

					Average	35%	2.93	2.80

					St dev	28.31%	0.77	0.75

## Results

**Figure 1:**
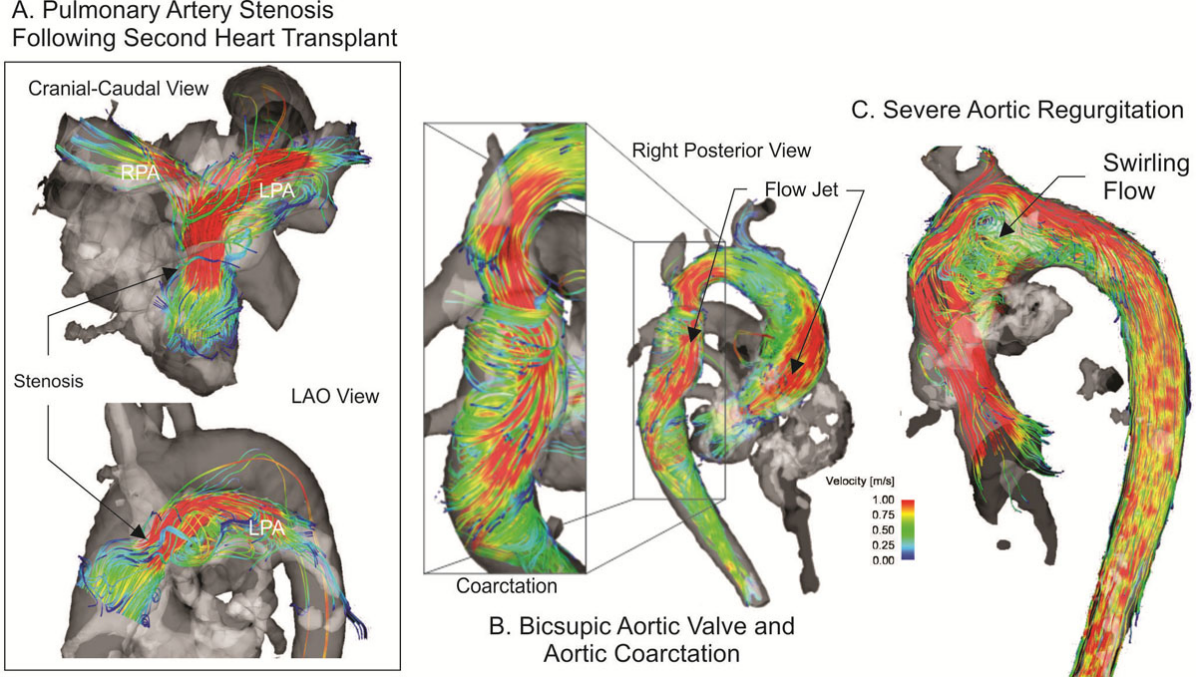
Velocity streamline flow representation in three clinical cases. A. Abnormal pulmonary flow in patient with pulmonary artery stenosis after two heart transplants. Note the helical flow and high velocities after the stenosis directed toward the left pulmonary artery (LPA) and decreased flow towards the right pulmonary artery (RPA). B. Deranged flow along the entire thoracic aorta secondary to bicuspid aortic valve and aortic coarctation. Note high velocity flow jet directed posteriorly with helix formation in the ascending aorta, and high velocity flow jet with helix formation distal to the coarctation. C. Flow abnormalities secondary to severe congenital aortic insufficiency. Note the high velocity systolic flow jet accompanied by swirling flow in the ascending aorta resulting from large volume aortic regurgitation.

Sixteen patients had clinical 4D flow MRI over 10 weeks. Clinical indications are reviewed in Table 1. The average 4D flow impact factor (scale 1-4) excluding and including 2D PC was 2.93 +/- 0.77 and 2.80 +/- 0.75, respectively. The average percent difference in quantitative data was 35 +/- 28%. One patient could not be evaluated secondary to aliasing. Average time for 4D flow post-processing was 88.5 +/- 22.5 min. Three clinical cases are shown in Figure 1.

## Conclusions

Including 4D flow MRI as part of clinical CMR workflow is feasible and has the potential to impact clinical assessment in multiple cardiovascular pathologies. The ability to evaluate flow throughout the acquired 3D volume retrospectively may reduce dependence on time-intensive 2D PC acquisitions while yielding accurate and efficient flow quantification.

## Funding

Grant support: NIH R01HL115828, NUCATS Dixon Award
